# Green Synthesis of Silver Nanoparticles, Their Characterization, Application and Antibacterial Activity ^†^

**DOI:** 10.3390/ijerph10105221

**Published:** 2013-10-21

**Authors:** Florence Okafor, Afef Janen, Tatiana Kukhtareva, Vernessa Edwards, Michael Curley

**Affiliations:** 1 Department of Biological and Environmental Sciences, Alabama A&M University, P.O. Box 1672, Normal, AL 35762, USA; E-Mail: afef.janen@gmail.com; 2 Department of Physics, Chemistry and Mathematics, Alabama A&M University, 4900 Meridian Street N., Normal, AL 35762, USA; E-Mails: tanja.kukhtareva@aamu.edu (T.K.); vernessa.edwards@aamu.edu (V.E.); michael.curley@aamu.edu (M.C.)

**Keywords:** biosynthesis, silver nanoparticles, plant extracts, cytotoxicity bacteria, antibacterial activity

## Abstract

Our research focused on the production, characterization and application of silver nanoparticles (AgNPs), which can be utilized in biomedical research and environmental cleaning applications. We used an environmentally friendly extracellular biosynthetic technique for the production of the AgNPs. The reducing agents used to produce the nanoparticles were from aqueous extracts made from the leaves of various plants*.* Synthesis of colloidal AgNPs was monitored by UV-Visible spectroscopy. The UV-Visible spectrum showed a peak between 417 and 425 nm corresponding to the Plasmon absorbance of the AgNPs. The characterization of the AgNPs such as their size and shape was performed by Atom Force Microscopy (AFM), and Transmission Electron Microscopy (TEM) techniques which indicated a size range of 3 to 15 nm. The anti-bacterial activity of AgNPs was investigated at concentrations between 2 and 15 ppm for Gram-negative and Gram-positive bacteria. *Staphylococcus aureus* and *Kocuria rhizophila*, *Bacillus thuringiensis* (Gram-positive organisms); *Escherichia coli*, *Pseudomonas aeruginosa*, and *Salmonella typhimurium* (Gram-negative organisms) were exposed to AgNPs using Bioscreen C. The results indicated that AgNPs at a concentration of 2 and 4 ppm, inhibited bacterial growth. Preliminary evaluation of cytotoxicity of biosynthesized silver nanoparticles was accomplished using the InQ™ Cell Research System instrument with HEK 293 cells. This investigation demonstrated that silver nanoparticles with a concentration of 2 ppm and 4 ppm were not toxic for human healthy cells, but inhibit bacterial growth.

## 1. Introduction

The application of nanoscale materials structures, usually ranging from 1 to 100 nanometers (nm), is an emerging area of nanoscience and nanotechnology. Nanomaterials may provide solutions to technological and environmental challenges in the areas of solar energy conversion, catalysis, biology, biomedical science, and water treatment. Nanoparticles possess very high surface to volume ratios. This property can be utilized in the scientific fields, where high surface area is needed. As an example, in the catalytic industry, some nanoparticles have actually proven to be good catalysts [1]. Moreover, the nanoparticles show bactericidal effects. These essential qualities have been attributed to silver nanoparticles (AgNPs). Therefore, in nano-biotechnological research, AgNPs have received significant attention because of their unique physical, chemical, biological properties, and because of their applicability in electronics, optics and medicine [2,3,4,5,6,7,8,9]. 

Moreover, silver nanoparticles have different important applications: for example, they might be used as spectrally selective coatings for solar energy absorption, as an intercalation material for electrical batteries, and as optical receptors for biolabeling [10,11,12,13]. AgNPs are well known as inhibitory and antibacterial materials. However, resistance to antimicrobial agents by pathogenic bacteria has emerged in recent years. This is a major challenge for the health care industry, and has been widely studied [14,15,16,17,18,19,20,21,22,23,24]. 

Chemical and physical methods may successfully produce pure, well-defined nanoparticles, but these techniques are more expensive, energy consuming and potentially toxic to the environment. In this paper, we are considering an environmentally friendly technique to produce AgNPs using extracellular biosynthesis. Biosynthetic methods can employ either microorganism cells or plant extract for nanoparticles production. An exciting branch of biosynthesis of nanoparticles is the application of plant extracts to the biosynthesis reaction. Recently, the green processes for the synthesis of nanoparticles are evolving into an important branch of nanotechnology [25,26,27,28,29,30,31,32,33]. The choice of plants in the present paper has been made based on their medical applications [34,35,36]. 

In this paper we present a rapid method for nanoparticles production using plant leaves extracts, their characterization and their inhibitory effect against Gram-negative and Gram-positive bacteria. In addition, we are submitting the preliminary results of cytotoxicity of biosynthesized nanoparticles for Human Embryonic Kidney 293 cells (HEK 293).

## 2. Materials and Methods

The plants selected for silver nanoparticles production are listed in Table 1: 

**Table 1:** Selected plants used for silver nanoparticles synthesis.

Scientific name	Common name
*Actaea racemosa*	Black cohosh
*Magnolia grandiflora*	Magnolia
*Aloe sp.*	“Tingtinkie”
*Eucalyptus* *angophoroides*	Eucalyptus
*Sansevieria trifasciata*	Mother-in-laws’ tongue
*Impatiens balsamina*	Rose Balsam
*Pelargonium graveolens*	Geranium

For nanoparticles production we used plant leaves extracts as reducing agents for silver nitrate salt AgNO_3_ (Carolina Biological Supplies, Burlington, NC, USA) to reduce nanoparticles. Ten grams of leaves were washed and finely cut, boiled in 50 mL of sterile distilled water for 5 min, 3 mL of the filtrated plant water extract was added to a heated 75 °C AgNO_3_ solution (50 mL of 10^−^^3^ M of AgNO_3_) [37,38]. The solution was then tested using a UV-Visible spectrophotometer (Varian, CARY 3E). The plant leaves extracts were also prepared using 10 g of finely cut plant leaves placed in 100 mL ethyl alcohol (C_2_H_5_OH) for 24 h.

Transmission Electron Microscopy analysis (TEM) was performed using a FEI Tecnai G2 Spirit Transmission Electron Microscope (Ohio State University, Dayton, OH, USA). Atomic Force Microscopy (AFM) analysis was completed using a Solver Scanning Probe Microscope (Solver P47H, Moscow, Russia) by using full contact and semi-contact methods. 

The antibacterial activity of silver nanoparticles was evaluated against three Gram negative bacteria (*Escherichia coli* ATCC 25922, *Salmonella typhimurium* ATCC 14028, and *Pseudomonas aeruginosa* ATCC 15442) and three Gram positive bacteria (*Staphylococcus aureus* ATCC 6538, *Kocuria rhizophila* ATTC 9341, and *Bacillus thuringiensis* ATCC 10792)*.* The microbiological media used in the study was supplied by Becton, Dickinson and Company (Franklin Lakes, NJ, USA). Growth and cytotoxicity analysis was performed using Bioscreen C (Automated Microbiology Growth Analysis System) manufactured in Finland by OY Growth Curves CE. 

To study growth of bacteria in broth culture, 5 mL of bacterial culture were placed in a 250 mL flask containing the 200 mL of Bacto Nutrient broth. The culture was then placed in a Lab-Line^®^ Incubator-Shaker by Orbit at a temperature of 37 °C, shaking at 175 rpm for 24 h. A 1:10 mixture of the 24 h bacterial growth and sterile physiological saline water was prepared. An aliquot of 100 µL from each solution was placed into different wells of the Bioscreen C. The first column wells served as controls and had no silver nanoparticles. Aliquots of 10 µL and 20 µL of different concentrations of silver nanoparticles were added to the samples. Growth rate was determined by measuring turbidity using the Bioscreen C for a total of 24 h at regular intervals (15 min). 

In order to determine the dose response effect of the silver nanoparticles, both Gram-positive and Gram-negative bacteria were exposed to two different concentrations of silver nanoparticles (2 ppm and 4 ppm). An optical density (OD) curve was generated based on the turbidity measurement over a period of 24 h. During the experiment, data were exported to a PC in MS Excel. Microbiological calculations were generated directly to MS Excel sheets automatically. Measurements were then processed to generate microbiological growth curves, plotting turbidity *vs.* time [39].

The cytotoxicity evaluation was conducted using the InQ Plus bench top cell research system using HEK 293 cell. HEK 293 cells were divided and plated into InQ Cell Growth Cassettes at the density of 3 × 10^5^cells/mL in a total volume of 8 mL per well (16 mL) of 10% FBS DMEM. Two mL of AgNPs solution with various concentrations for several experiments in one cassette, and control solution (leave extract, pure solution of AgNO_3_) in second cassette has been injected. Three data points were selected and saved per side. Images were collected at 600 s intervals during the 48 h experiments. The untreated cells and plant extract solutions without AgNPs were used as negative control, while DMSO was used as positive control.

## 3. Results and Discussion

### 3.1. Nanoparticles Production and Characterization

**Figure 1:**
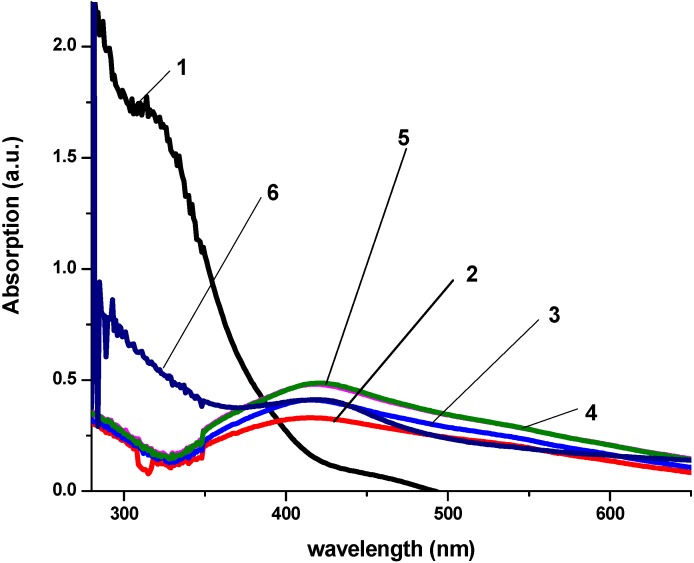
Kinetic of reaction process of AgNPs production using *Magnolia grandiflora* water extract as reduction agent. (**1**) *Magnolia* leaves water extract in sterile distilled water. (**2**) AgNO_3_(10^−3^ M) + *Magnolia* aqueous extract (3 mL) after 3 min of reaction. (**3**) AgNO_3_ (10^−3^ M) + *Magnolia* water extract (3 mL) after 5 min of reaction. (**4**) AgNO_3_ (10^−3^ M) + Magnolia water extract (3 mL) after 10 min of reaction. (**5**) AgNO_3_ (10^−3^ M) + *Magnolia* water extract (3 mL) after 20 min of reaction. (**6**) Commercial Ag Colloidal solution—10 ppm concentration.

**Figure 2:**
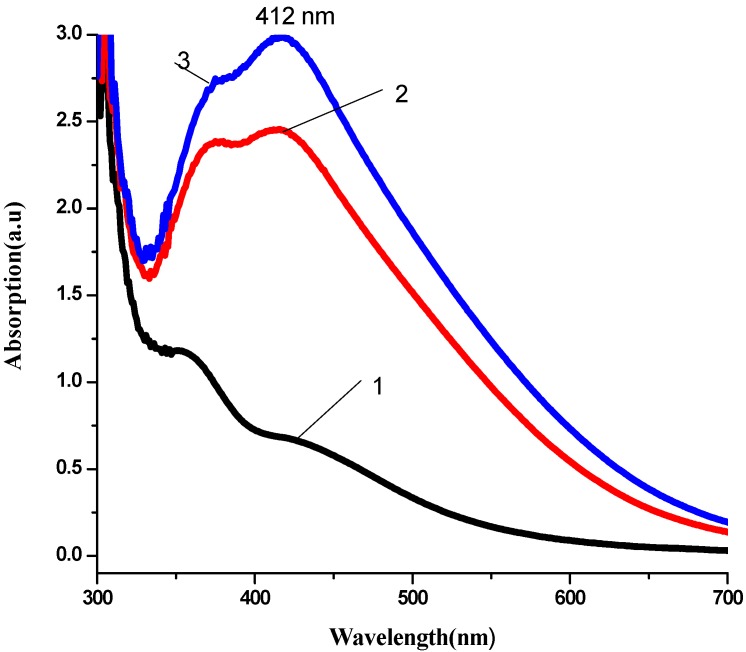
Kinetic of reaction process of AgNPs production using *Geranium* leaf water extract as reduction agent (**1**) 1 min after reaction, (**2**) 3 min after reaction, (**3**) 5 min after reaction.

**Figure 3:**
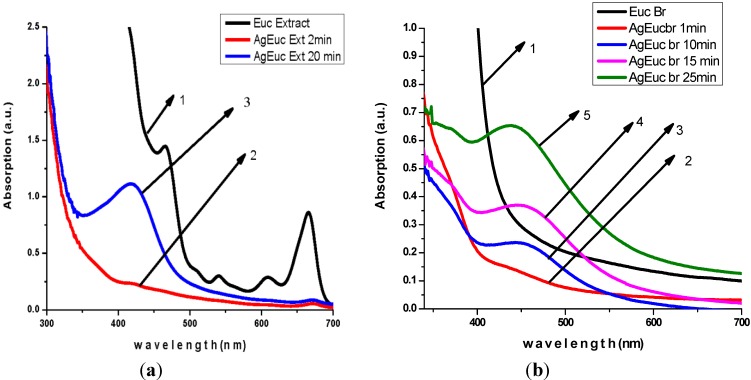
Kinetic of reaction process of AgNPs production using *Eucalyptus* ethanol extract (**a**) and water extract (**b**) as reduction agent.

The production of AgNPs was monitored using UV-Visible spectrometer. Kinetics of the reaction AgNPs production process are presented in Figure 1, Figure 2 and Figure 3. 

(**a**) 1: 2 min after reaction; 2: 1 min after reaction; 20 min after reaction; 3: 10 min after reaction;

(**b**) 1: 1 min after reaction; 2: 10min after reaction; 3: 15 min after reaction; 4: 25min after reaction; 5: 25 min after reaction.

The production of AgNPs by *Aloe* “Tingtinkie” leaves aqueous extract*, Actaea racemosa* (black cohosh), *Sansevieria trifasciata* and *Impatiens scapiflora leaves* aqueous extract and ethanol extracts demonstrated similar UV-Visible spectra.

The analysis of UV-Visible spectroscopy data showed an appearance of surface plasmon resonance peak (SPR) at the 417–430 nm wavelength range, which corresponds to AgNPs production. AgNPs absorb radiation intensely at a wavelength of 400 nm due to the transition of electrons. The exact mechanism of the extracellular biosynthesis of metal NP in not well understood. It was hypothesized that NADH coenzyme was working as an electron shuttle to neutralize Ag^+^ ion [40,41] .

**Figure 4:**
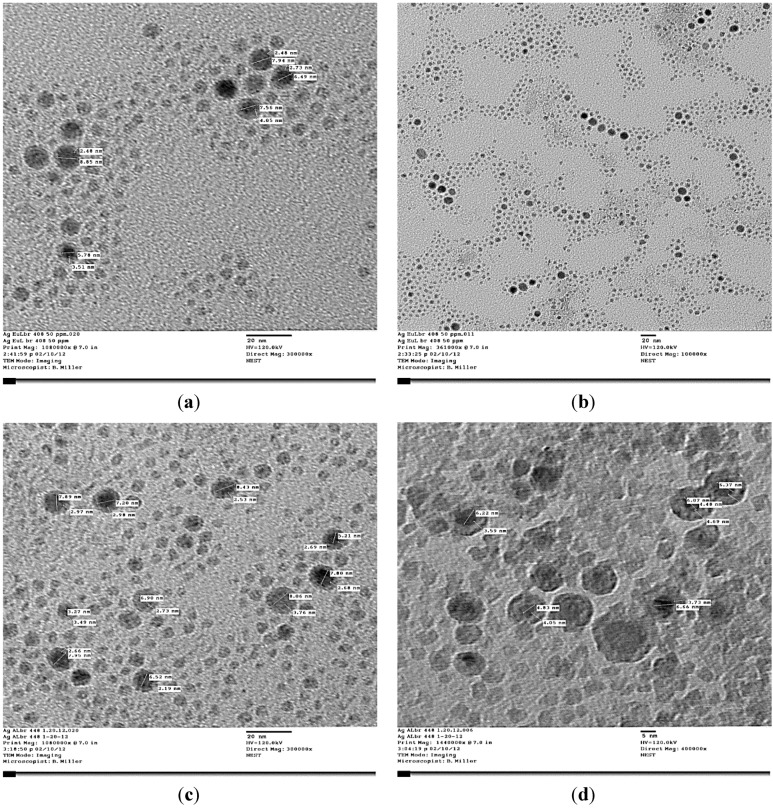
TEM Images of AgNPs produced by *Eucalyptus* (**a**); (**b**) and *Aloe* plant (**c**), (**d**).

**Figure 5:**
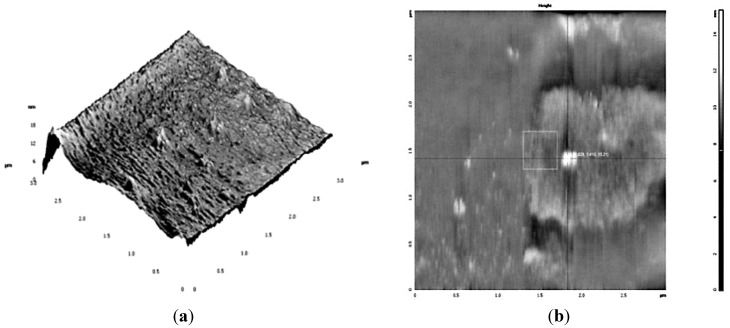
AFM images of AgNPs produces by *Geranium* water extract. (**a**) 3-D image of Ag nanoparticles produced by *Geranium* water extract; (**b**) Estimation of nanoparticles size by decreasing the scanning range (3 µm), the size of AgNPs as shown on the image is ~15.2 nm.

The AgNPs was characterized by Transmission Electron Microscopy (TEM) and Atomic Force Microscopy (AFM) (Figure 4 and Figure 5). Transmission Electron Microscopy (TEM) results showed particles with spherical shape surrounded by biological molecules, which prevent AgNPs from aggregation. As shown in Figure 4 the average size of AgNPs is in the 3–9 nm range. 

### 3.2. Antibacterial Activity of Silver Nanoparticles

**Figure 6:**
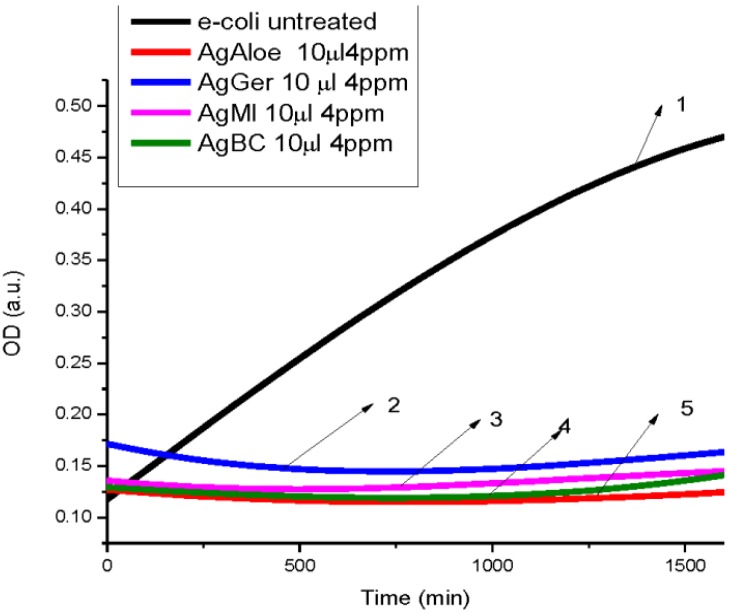
Dependence optical density on time of *E. coli* (**1**) Untreated *E. coli*. (**2**) Treated with 10 µL, 4 ppm AgNPs produced by *Geranium*. (**3**) Treated with 10 µL, 4 ppm AgNPs produced by *Magnolia*. (**4**) Treated with 10 µL, 4 ppm AgNPs produced by black cohosh. (**5**) Treated with 10 µL, 4 ppm AgNPs produced by *Aloe*.

The antibacterial activity of silver nanoparticles using select plants was found to exert inhibitory effects on different species of bacteria: three Gram negative bacteria and three Gram positive bacteria. The silver nanoparticles synthesized from aloe, geranium, magnolia and black cohosh extracts at a concentration of 4 ppm, all inhibited the growth of *E. coli* based on the OD readings as compared to the control. The control wells without AgNPs showed OD absorption units at 0.46, while the test wells showed OD units between 0.12 and 0.17 indicating little or no growth (Figure 6). Aloe extract nanoparticles showed the highest antibacterial activity followed by black cohosh and geranium nanoparticles with the lowest inhibition. These results are in agreement with previous studies [42] indicating that aloe-produced nanoparticles have a high inhibitory growth on *E. coli* at low concentrations. 

**Figure 7:**
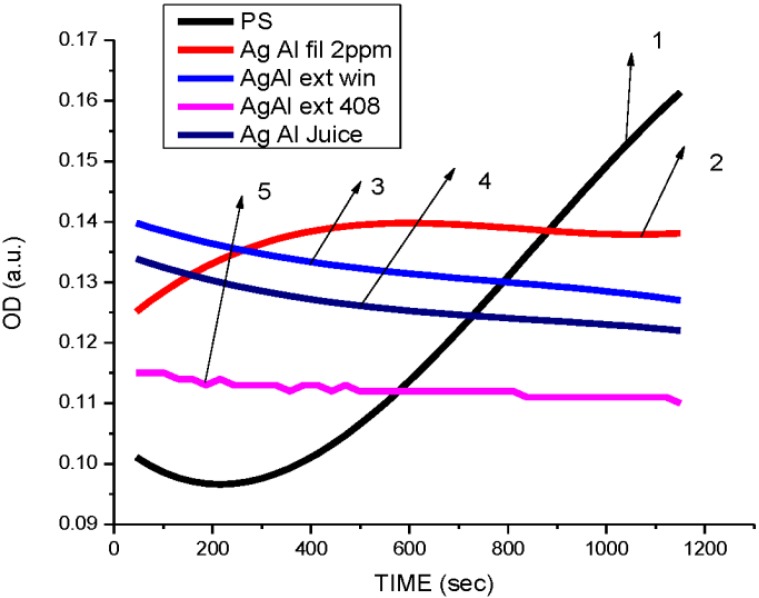
Dependence optical density on time of *Pseudomonas—*Gram-negative bacteria (**1**) Untreated *Pseudomonas*. (**2**) Treated with 10 µL, 2 ppm AgNPs reduced by *Aloe* leaves water extract. (**3**) Treated with 10 µL, 4 ppm AgNPs reduced by *Aloe* leaves ethanol extract. (**4**) Treated with 10 µL, 4 ppm AgNPs reduced by *Aloe* juice. (**5**) Treated with 10 µL, 4 ppm AgNPs reduced by *Aloe* leaves extract (AgNP size 2–8 nm, Figure 5(c,d)).

**Figure 8:**
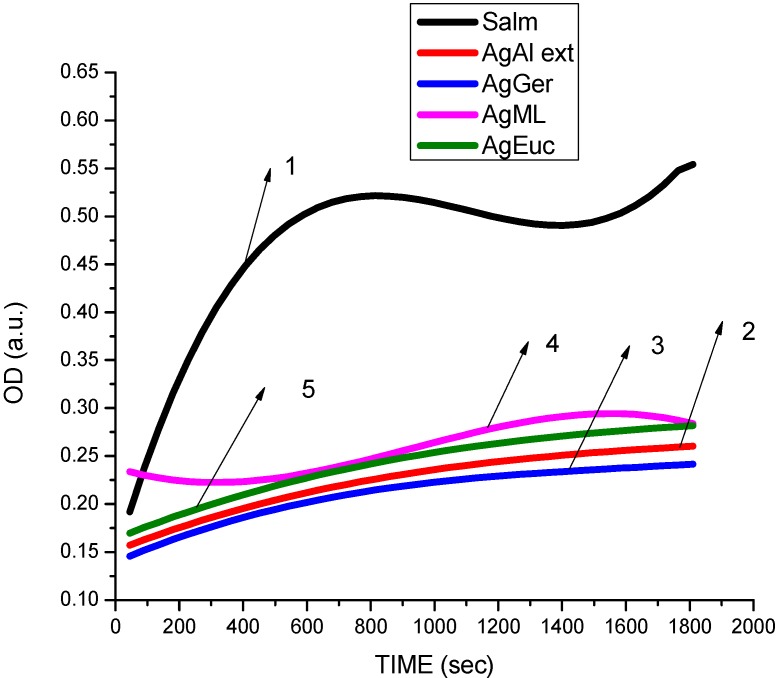
Dependence optical density on time of *Salmonella—*Gram-negative bacteria treated with AgNPs reduced by various plants leaves. (**1**) Untreated *Salmonella*. (**2**) Treated with 10 µL, 4 ppm AgNPs produced by aloe leaves ethanol extract. (**3**) Treated with 10 µL, 4 ppm AgNPs produced by *Geranium* leaves water extract. (**4**) Treated with 10 µL, 4 ppm AgNPs produced by *Magnolia* leaves water extract*.* (**5**) Treated with 10 µL, 4 ppm AgNPs produced by *Eucalyptus* leaves water extract.

**Figure 9:**
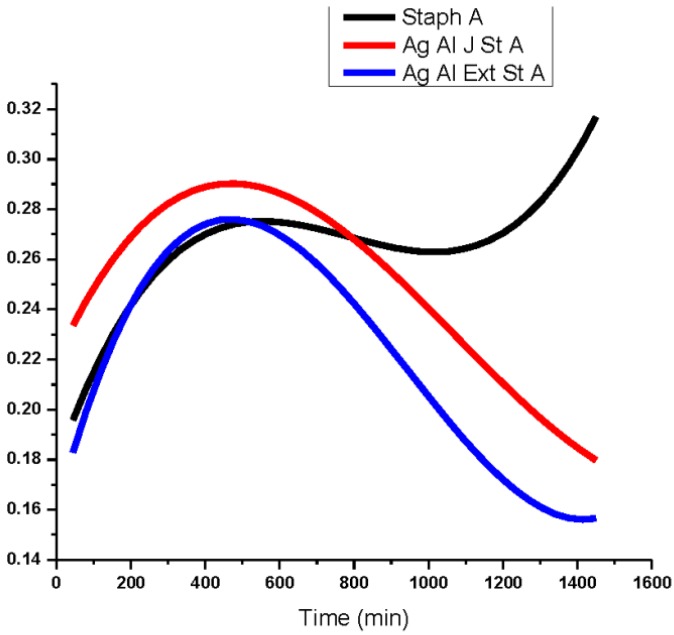
Dependence optical density on time of *Staphylococcus aureus* Gram-positive bacteria treated with AgNps reduced by aloe plant juice (red) and ethanol extract (blue).

According to Gogoi *et al.* [43], the cell surface of *E. coli* is negatively charged and thus Ag^+^ easily interacts with cell membranes, thus disabling their function. The silver nanoparticles from aloe extracts, at 4 ppm, had a bacteriostatic effect on *Pseudomonas* (Figure 7) compared to the untreated species. Similar results were obtained in the case of *Salmonella* where aloe extract—produced nanoparticles showed the highest antibacterial activity at a concentration of 2 ppm compared to the geranium, eucalyptus, and magnolia at 4 ppm. Aloe-produced silver nanoparticles inhibited the growth of *Salmonella typhimurium* (Figure 8) more than the growth of *E. coli*. Antibacterial activity of aloe silver nanoparticles on *Staphylococcus* (Figure 9) was less pronounced and antibacterial activity was delayed in comparison. Researchers [44,45] have reported similar results, in that DNA of *Staphylococcus aureus* cells loses its replication ability after 6 h of exposure to 50 µg/mL AgNPs. The delay may also be due to the presence of thick peptidoglycan and teichoic acids in the Gram-positive bacterial cell wall.

**Figure 10:**
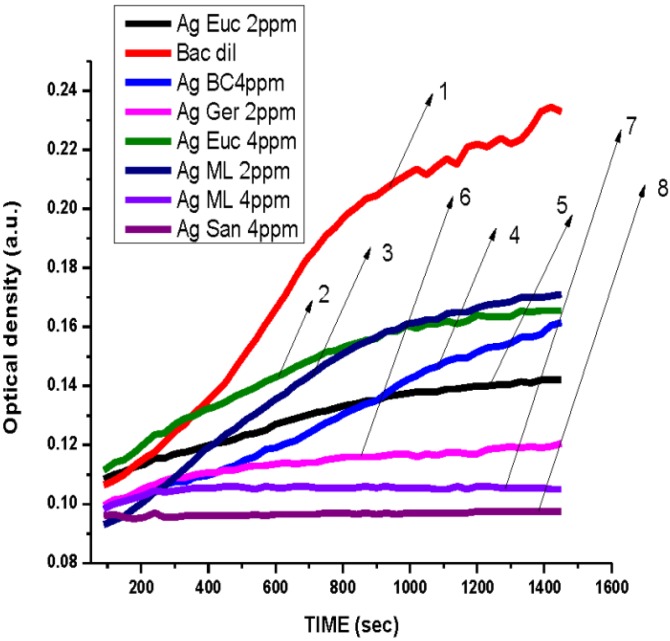
Dependence optical density on time of *Bacillus—*Gram-positive bacteria treated with AgNPs reduced by various plants leaves, (**1**) *Bacillus*—no treatment, (**2**) Treated with 10 µL, 4 ppm AgNPs produced by *Eucalyptus* leaves water extract*.* (**3**) Treated with 10 µL, 2 ppm AgNPs produced by *Magnolia* leaves water extract. (**4**) Treated with 10 µL, 4 ppm AgNPs produced by black cohosh extract. (**5**) Treated with 10 µL, 2 ppm AgNPs produced by *Eucalyptus* leaves water extract. (**6**) Treated with 10 µL, 2 ppm AgNPs produced by *Geranium* leaves water extract. (**7**) Treated with 10 µL, 4 ppm AgNPs produced by *Magnolia* leaves water extract. (**8**) Treated with 10 µL, 4 ppm AgNPs produced by *Sansevieria* leaves extract.

The antibacterial effect of the silver nanoparticles from the test plant extracts was evaluated against *Bacillus* (Figure 10) at concentrations of 2 and 4 ppm. The AgNPs reduced by *Magnolia* and by *Geranium* did not show a direct dose-response relationship with *Bacillus thuringensis*, since growth inhibition appeared to decrease at 4 ppm. Also, as shown in Figure 10, nanoparticles reduced by *Sansevieria* demonstrated a considerable growth inhibition of *Bacillus.* The silver nanoparticles prepared by *Geranium* inhibited the growth of *Bacillus* at a concentration of 2 ppm. Although growth of *Kocuria rhizophila* was inhibited on exposure to AgNPs from *Eucalyptus* extracts, inhibition was more pronounced at 4 ppm. 

**Figure 11:**
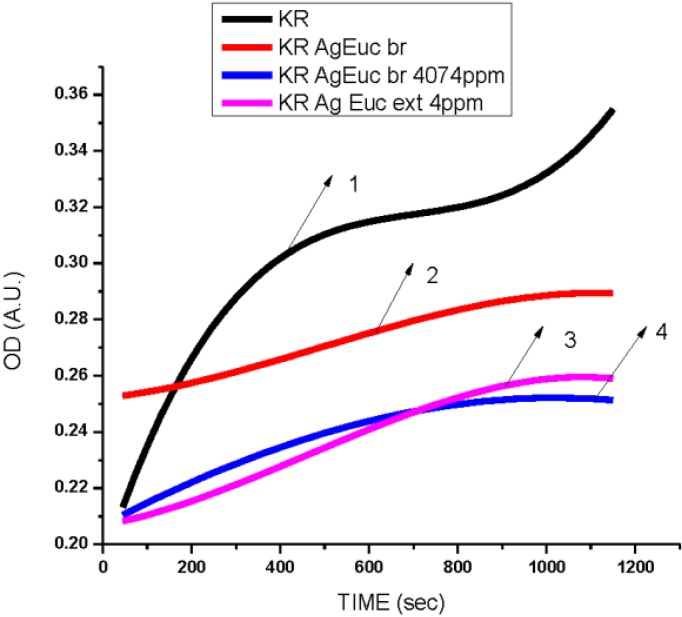
Dependence optical density on time of *Kocuria rhizophila—*Gram-positive bacteria treated with AgNPs reduced by *Eucalyptus* plants leaves. (**1**) untreated *Kocuria rhizophila*. (**2**) Treated with 10 µL, 4 ppm AgNPs produced by *Eucalyptus* leaves water extract. (**3**) Treated with 10 µL, 4 ppm AgNPs produced by *Eucalyptus* leaves water extract (AgNPs size 2–8 nm, Figure 5(a,b)) (**4**) Treated with 10 µL, 4 ppm AgNPs produced by *Eucalyptus* leaves ethanol extract.

The overall results indicated that AgNPs reduced using different plants extracts showed antibacterial activity at doses of 2 and 4 ppm towards the Gram-positive and Gram-negative test bacteria (Figure 6, Figure 7, Figure 8, Figure 9, Figure 10 and Figure 11) Also, the results of our antibacterial study revealed that AgNps reduced by aloe extract showed the best inhibitory activity against the tested bacteria. Our speculation is that the aloe-produced AgNPs may contain some bioactive molecules (quinones, other aromatic compounds) from aloe and this combination enhanced the inactivation or growth inhibition of the bacteria species [42]. In effect, the high antimicrobial effects of the aloe-produced silver nanoparticles may be due to a combination of the AgNPs and aloe materials. We are currently studying this.

The plant extract-produced AgNPs exhibited high inhibitory effects against *E. coli* and *Salmonella* while moderate activity was observed for *Pseudomonas aeruginosa, Bacillus subtilis* and *Kocura rhizophila*. Silver nanoparticles were found to be comparatively less active in killing *Staphylococcus aureus*. This implies that the antibacterial sensitivity of the gram-positive *Staphylococcus aureus* was greatly lower than that of the gram-negative *E. coli*. This is possibly due to the thickness of the peptidoglycan layer of *Staphylococcus aureus* which protects against toxins and chemicals [46].

Despite their extensive use, the antibacterial mechanism of the silver nanoparticles is still unclear. However it has been reported [47] that when silver nanoparticles are attached to the surface of the cell membrane, the respiratory function and permeability of the bacterial cells become unstable. Other studies suggest that when bacteria are treated with silver ions, DNA tends to lose its ability to replicate [48]. Also the cell wall structure of Gram negative bacteria facilitates Ag^+^ access to the cytoplasmic membrane compared to the Gram positive bacteria. 

### 3.3. Cytotoxicity of Biosynthesized Noble Nanoparticles on Healthy Human Cells

We tested the cytotoxicity of Ag Nanoparticles produced using extracts of *Aloe*, *Magnolia* leaves and *Eucalyptus* leaves at concentrations of 2 ppm, 4 ppm, 15 ppm on Human Embryonic Kidney 293cells—HEK293 using the automated InQ Plus equipment.

**Figure 12:**
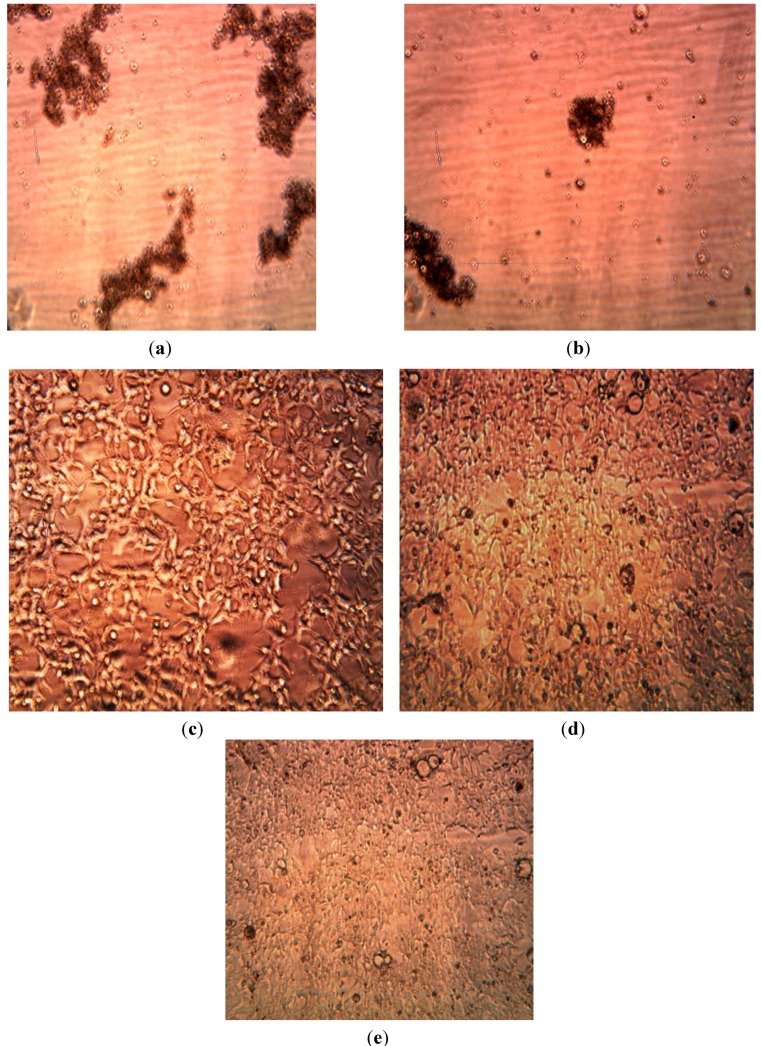
Images of HEK 293 cells. (**a**) 6 h after *Aloe* ethanol extract injection. (**b**) 48 h after *Aloe* ethanolic extract injection. (**c**) 6 h after *Aloe* extract-produced AgNPs, (**d**) after 36 h, (**e**) after 48 h.

**Figure 13:**
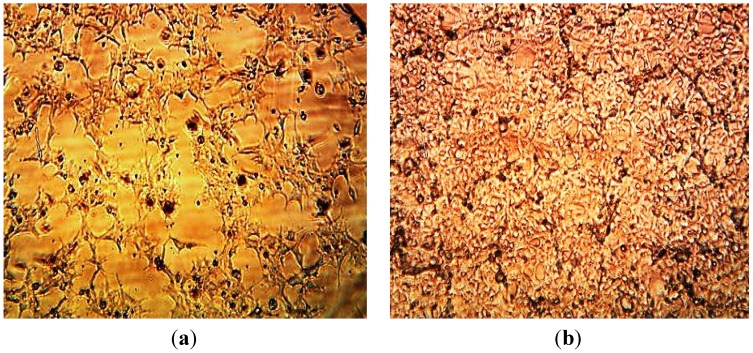
Images of HEK 293 cells: (**a**) 6 h after injection of 4 ppm AgNPs reduced using *Eucalyptus* extract. (**b**) 48 h after injection of 4 ppm AgNPs.

**Figure 14:**
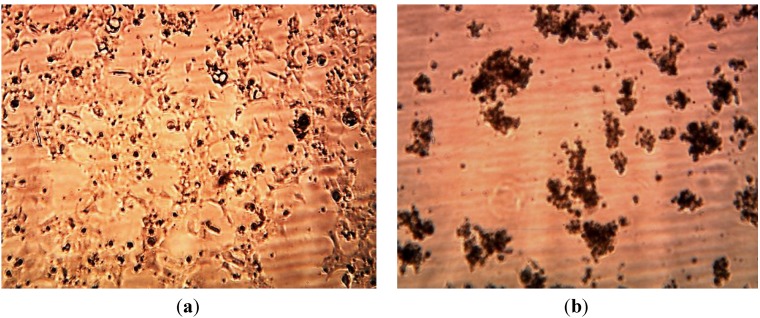
Images of HEK 293 cells: (**a**) 6 h after injection of 4 ppm AgNPs reduced by *Eucalyptus extracts* (**b**) Images of HEK 293 cells: 48 h after injection of 15 ppm AgNPs reducing by *Eucalyptus ethanol extract*.

The cassette containing the growing cells was divided in two parts. In real time we were able to compare the influence of the biosynthesized nanoparticles on human cell growth and control solutions. Figure 12, Figure 13 and Figure 14 below show our observations indicating that bio-produced AgNPs at concentrations of 2–4 ppm were not cytotoxic to human embryonic kidney cells (HEK 293). From available literature, silver nanoparticles (AgNPs) may exhibit toxic or nontoxic effects on various cells depending on their size, concentration and surface properties [49,50,51,52]. Bhakat *et al.* [53] found that their silver nanoparticles were toxic to HEK 293 cells. In the present study we found that biosynthesized AgNPs appeared to be non-toxic at 2–4 ppm concentrations to cultured human cells (HEK 293 cells) but were toxic to both Gram positive and Gram negative microorganisms. The non-toxicity of the biosynthesized nanoparticles to HEK 293 cells was probably due to the low concentration used in this study; this is similar to the report of Kawata *et al.* [51] They evaluated the *in vitro* toxicity of AgNPs at non-cytotoxic doses (<0.5 mg/L) in human hepatoma cell line, HepG2 and observed that the AgNPs actually accelerated cell proliferation at low doses due to induction of genes associated with cell cycle progression. We did not observe cell proliferation in the present study. Detailed and comprehensive dose-response studies need to be conducted in order to fully understand the effects of silver nanoparticles on human cells before these particles can be used in disease treatment and diagnosis.

## 4. Conclusions

The results showed that substantial amounts of silver nanoparticles (AgNPs) were produced and that this was verified using UV-visible Spectroscopy. We observed appearance of a Surface Plasmon Resonance (SPR) peak, which corresponds to the silver nanoparticles—417 nm in 1 min. In conclusion and based on our results, extracts of plant leaves produce usable silver nanoparticles. These nanoparticles inhibit the growth of both Gram-negative and Gram-positive bacteria, while based on preliminary evaluation of cytotoxicity of biosynthesized silver nanoparticles on HEK 293 cells healthy human cells were not adversely affected. This investigation demonstrated that bio-produced silver nanoparticles at concentrations of 2 ppm and 4 ppm are not cytotoxic for human healthy cells but inhibit bacteria growth. Further studies will be carried out to determine the minimum inhibitory concentration of AgNPs for bacterial growth, and to investigate the cytotoxicity of biosynthesized AgNPs on cancerous and other healthy cells.
